# Short Hospital Stay after Laparoscopic Colorectal Surgery without Fast Track

**DOI:** 10.1155/2012/260273

**Published:** 2012-11-19

**Authors:** Stefan K. Burgdorf, Jacob Rosenberg

**Affiliations:** Department of Surgical Gastroenterology, Herlev Hospital, University of Copenhagen, Herlev Ringvej 75, 2730 Herlev, Denmark

## Abstract

*Purpose*. Short hospital stay and equal or reduced complication rates have been demonstrated after fast track open colonic surgery. However, fast track principles of perioperative care can be difficult to implement and often require increased nursing staff because of more concentrated nursing tasks during the shorter hospital stay. Specific data on nursing requirements after laparoscopic surgery are lacking. The purpose of the study was to evaluate the effect of operative technique (open versus laparoscopic operation), but without changing nurse staffing or principles for peri- or postoperative care, that is, without implementing fast track principles, on length of stay after colorectal resection for cancer. *Methods*. Records of all patients operated for colorectal cancer from November 2004 to December 2008 in our department were reviewed. No specific patients were selected for laparoscopic repair, which was solely dependent on the presence of two specific surgeons at the same time. Thus, the patients were not selected for laparoscopic repair based on patient-related factors, but only on the simultaneous presence of two specific surgeons on the day of the operation. *Results*. Of a total of 540 included patients, 213 (39%) were operated by a laparoscopic approach. The median hospital stay for patients with a primary anastomosis was significantly shorter after laparoscopic than after conventional open surgery (5 versus 8 days, *P* < 0.001) while there was no difference in patients receiving a stoma (10 versus 10 days, ns), with no changes in the perioperative care regimens. Furthermore there were significant lower blood loss (50 versus 200 mL, *P* < 0.001) and lower complication rate (21% versus 32%, *P* = 0.006) in the laparoscopic group. *Conclusion*. Implementing laparoscopic colorectal surgery in our department resulted in shorter hospital stay without using fast track principles for peri- and postoperative care in patients not receiving a stoma during the operation. Consequently, we aimed to reduce hospitalisation without increasing cost in nursing staff per hospital bed. Length of stay was not reduced in patients receiving a stoma pointing at this group for specific intervention in the future. Furthermore, the complication rate was reduced in the laparoscopic group.

## 1. Introduction

Colorectal cancer (CRC) is a common disease in the western world. Even though therapies like radio-, chemo- and newer immune-therapies have evolved and improved during the last decades, the prognosis for end-stage disease is still poor and surgery remains the only curative therapy [1]. Prognosis associated with CRC have improved due to earlier detection of malignancy, better and more radical surgical techniques and more effective adjuvant therapies, but there is still room for improvement.

Short hospital stay and equal or reduced complication rates have been demonstrated after fast track open colonic surgery [2]. Fast track principles of peri- and postoperative care, however, often require some changes in the surgical departments. As a consequence of more concentrated nursing tasks during the shorter hospital stay, increased nursing requirements per bed is required [3]. Implementation of these major changes has in many cases been problematic and unsuccessful [4]. However, in a gynaecological department enhanced recovery has been introduced without compromising the workload of the nurse staffing [5].

Laparoscopic colectomy was first reported by Jacobs et al. in 1991 [6] and since then minimally invasive gastrointestinal surgery has become more popular. The surgical trauma from laparoscopic surgery is significantly reduced compared with conventional open surgery. In spite of obvious advantages in laparoscopic surgery there is still an ongoing debate on complication rates, hospitalisation, nurse staff requirements, blood loss, radicality, conversion rate, learning curve and implementation difficulties, mortality, and overall survival in minimally invasive techniques compared with conventional open techniques [7]. Most studies have shown shorter hospitalisation for patients undergoing laparoscopic colonic surgery compared with open colonic surgery, but most of the available studies have either not been formally controlled randomised studies or not controlling perioperative care regimens. However, a recent randomised controlled 9-center trial concludes that the optimal perioperative treatment after segmental colectomy is laparoscopic approach in a fast track setting [7]. Data on nursing requirements after laparoscopic surgery are lacking. Therefore, possibly by only changing the operative procedure, a shorter hospitalisation may be obtained after laparoscopic surgery, without changing nurse staffing or principles for perioperative care, that is, without implementing fast track surgery principles.

The aim of this paper was to show the effect of laparoscopic surgery per se on hospital stay and complication rates after colonic and rectal resections in a retrospective controlled study in a setting where nurse staffing was not changed, but only changing the surgical procedures to a minimally invasive approach. 

## 2. Methods

### 2.1. Patients

From the first laparoscopic operation in November 2004 through December 2008, 213 patients underwent laparoscopic and 327 patients underwent open surgery for CRC in the Department of Surgical Gastroenterology, Gentofte University Hospital, Copenhagen, Denmark. No specific patients were selected for laparoscopic repair, which was only dependent on the presence of two specific surgeons that both had to be working on the same day. If these two surgeons were available then the operation was laparoscopic and if not it was open. Thus, the selection procedure was based on logistics and not on patient factors. If possible for logistic reasons the patients were offered laparoscopic approach. Records of all patients going through colonic and/or rectal resections in the study period were reviewed. Only patients with verified colonic or rectal carcinomas who underwent elective surgery were included in this study. 

The patients were all admitted to the same surgical department. Regardless of surgical approach they all received the same peri- and postoperative care by the same nurse staffing and the same doctors were doing the ward rounds. Fast track principles of peri- and postoperative care had not been implemented in the department. No certain criteria were used for discharge but was solely based on the doctors doing the ward rounds and was not dependent on the surgical approach.

### 2.2. Data Collection

All patient records were reviewed and all data extracted independently by two researchers and if inconsistencies consensus were made. The data retained from the patient records were demographics: sex, age, height, weight, and the American Society of Anaesthesiology (ASA) class; and parameters related to the operation: diagnosis, date of operation, primary anastomosis or stoma, blood loss, number of resected lymph nodes, and eventually conversion to open technique; and postoperative parameters: complications and duration of postoperative hospitalisation.

Since the study is a descriptive retrospective study neither consent nor ethical approval was obtained. This is in accordance with Danish law.

### 2.3. Implementation Setup

 The surgeons in our department had experience with laparoscopic appendectomy, cholecystectomy, and hernia repair but most of these surgeons had little or no experience with surgery of the colon and rectum. The colorectal surgeons in our department had limited knowledge about it and very little experience in minimally invasive procedures. One colorectal surgeon (limited laparoscopic experience) was introduced to laparoscopic techniques and performed the operations with the experienced laparoscopist (limited experience in colorectal surgery) as an assistant. The only selection of patients for laparoscopic procedure was that these two surgeons had to be working on the day of the operation. If these two surgeons were present, the patient scheduled for operation that day underwent laparoscopic repair. 

### 2.4. Statistical Analysis

A database with all relevant data was created in Microsoft Excel 2002 (Microsoft corp., USA). Statistical analyses were run on SPSS software version 15.0 (SPSS Inc., Chicago, USA). Differences between subgroups were analysed with the Mann-Witney or Fisher's Exact test where appropriate and *P* ≤ 0.05 was considered statistically significant. 

## 3. Results

A total of 540 patients were included in the study. The proportion of patients undergoing laparoscopic surgery was 39% (213/540). A comparison of the 213 laparoscopically treated patients with the 327 patients treated with conventional open surgery is shown in Table 1. Length of hospital stay was significantly shorter for patients going through laparoscopic surgery compared to open surgery (5 versus 8 days, *P* < 0.001). Numbers of the different surgical procedures are shown in Table 2. When comparing the open and laparoscopic groups there was no significant difference in sex, ASA class, and number of harvested lymph nodes. 

Overall, the patients were equally distributed between men and women with a median age of 72 years (range 36–94 years) and a mean BMI of 23.7 kg/m^2^ (range 13.9–42.3 kg/m^2^). Most of the patients (78%) were given a primary anastomosis, while the rest received a temporary or permanent stoma. No 30-day mortality occurred in the laparoscopic cohort, but one patient died before discharge, 38 days after the primary operation after anastomosis leakage, open reoperation, abscesses, and finally lung edema. The patients in the laparoscopic group were significantly younger (70 versus 72 years, *P* = 0.02) and had a higher BMI (23.9 versus 23.4 kg/m^2^, *P* = 0.02). 

**Figure 1:**
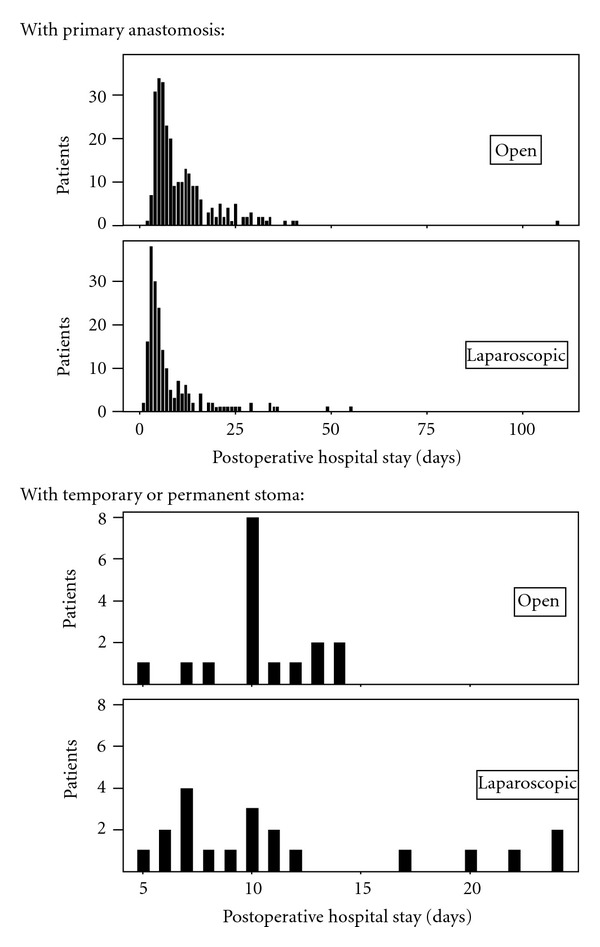
Histogram showing the frequencies of days of postoperative hospital stay after laparoscopic versus conventional open colonic and rectal resections for colorectal malignancies for patients with a stoma and for patients with primary anastomosis. For patients with a stoma there was no significant difference in postoperative hospital stay between laparoscopic and open surgery (median 10 versus 10 days, ns). For patients with primary anastomosis the median postoperative hospitalisation was significantly shorter (4 versus 8 days, *P* < 0.001) after laparoscopic repair.

With regard to perioperative differences, the laparoscopic group had significantly lower blood loss (50 versus 200 mL, *P* < 0.001) and equal proportion of primary anastomoses (86% versus 72%, ns). Postoperative comparison showed significantly lower complication rates (Table 1, *P* = 0.006) in the laparoscopic group. Complications were graded according to the Clavien-Dindo Classification of Surgical Complications [8]. Finally, the analyses of postoperative hospitalisation showed no difference between the two groups for the patients who received a stoma (10 versus 10 days, ns), but a significant difference in the larger subgroup of patients with primary anastomoses (4 versus 8 days, *P* < 0.001). These differences are also shown graphically in Figure 1.

 When comparing the laparoscopically treated patients who were given a stoma with those who received a primary anastomosis there was no significant difference in age (71 versus 69 years, *P* = 0.74), BMI (25.9 versus 23.6 kg/m^2^, *P* = 0.17), blood loss (175 versus 100 mL, *P* = 0.54), or number of resected lymph nodes (14 versus 15, *P* = 0.08), but the patients with a stoma had significantly longer postoperative hospital stay compared with the patients not receiving a stoma (10 versus 4 days, *P* = 0.001). There were no significant differences in age (*P* = 0.47), blood loss (*P* = 0.28), number of resected lymph nodes (*P* = 0.58), and postoperative hospital stay (*P* = 0.91) between the male and female patients in the laparoscopic group.

## 4. Discussion

This retrospective controlled study showed shorter hospital stay and lower complication rates after laparoscopic CRC surgery compared with conventional open surgery. The reduction in postoperative hospital stay was achieved without implementing any changes in the surgical ward regarding fast track principles for perioperative care. Thus, we were able to reduce hospitalisation without increasing nursing staff per hospital bed. 

The setup with a colorectal surgeon without laparoscopic experience assisted by a laparoscopically experienced “upper” GI surgeon was simple and successful. With a median postoperative hospital stay of 5 days, median 15 lymph nodes in the specimens, and 79% of cases without complications, we were able to produce results comparable with, or even better than our nationwide data [9, 10] and large multicentre controlled trials [11–18]. 

 Since the present results arise from a retrospective controlled study and not from a randomised controlled trial, and since the number of surgical procedures performed in the laparoscopic and open group is noncomparable, these results should be interpreted with caution. Nevertheless, they do indicate some of the advantages in laparoscopic CRC surgery and signify that laparoscopic CRC surgery may not be restricted to young and fit patients with low BMI and no comorbidities (low ASA class).

 We reported a quite high mortality rate in the conventional open group. Some of the patients in the conventional open group died of reasons not directly related to the surgery, but the exact reasons were not reported and as a consequence the high incidence cannot be explained.

The large randomised controlled trials all confirm that laparoscopy has a positive impact on postoperative restitution with earlier recovery of bowel function, less need for postoperative analgesics, and shorter postoperative hospital stay compared with conventional open surgery [12–20]. A prospective database study by Abraham et al. [20] resulted in the same short-term conclusions and even suggested lower operative mortality rates and better 3-year survival for patients treated laparoscopically. The benefits for long-term outcomes have not been confirmed in large randomised controlled trials [11–14, 17–19] which have shown no significant differences in operative mortality and long-term survival between laparoscopic and open surgery. Though for patients with Dukes D cancer laparoscopy has resulted in better survival than open surgery [19]. The effect of laparoscopy on mortality and survival is therefore, in spite of solid evidence on short-term outcomes, still questionable. All of the studies have, like the present, shown equal, or even better, lymph node harvesting with laparoscopic technique [22]. A more complete lymph node resection would theoretically improve long-term outcomes, but this has not been documented yet. A recent study has shown that the postoperative immune function remains highest in patients undergoing laparoscopic surgery with fast track care [23].

Several studies have dealt with the aspect of learning difficulties in laparoscopic colonic and rectal resections [24–28]. The surgical procedures are technically difficult, but with previous laparoscopic experience from appendectomies, cholecystectomies, and hernia repair the learning curve is not differing from that of conventional open colorectal surgery [25]. A theory could be that older surgeons initially may have difficulties in seeing 3-dimensional structures on a 2-dimensional screen, while younger upcoming surgeons have grown up with this from for example, computer games [29]. Most studies defined learning curves from intraoperative complications, rate of conversion to open surgery, and eventually number of harvested lymph nodes and operating time. Adequate learning occurred after 5–80 interventions [24–28, 30]. The reasons for the wide spread in these results could be that the studies were based on a few surgeons and these results are of course very much dependent on the skills of the specific surgeon. In one study with three surgeons the time for adequate learning ranged from 5–17 interventions [24]. Based on these studies and our own experiences we believe that laparoscopic colonic and rectal resections can be learned as quickly, effectively, and safely as conventional open resections.

## 5. Conclusion

Implementing laparoscopic colonic and rectal resection for colorectal malignancies in our department resulted, for patients with primary anastomoses, in shorter hospital stay compared with conventional open surgical technique. The implementation of laparoscopic colorectal surgery was done without implementing fast track principles for perioperative care. This study confirms that laparoscopic CRC surgery can be implemented successfully. The short-term outcomes after laparoscopic CRC surgery are superior to conventional open surgery. The long-term effects have to be confirmed in large randomised controlled trials, but do not seem worse than after open repair. A part of our success with laparoscopic repair concerning length of hospital stay could theoretically be patient, surgeon, or nursing staff biased. Therefore, future studies should evaluate the effect of laparoscopic versus open surgery in a blinded trial and without implementing fast track principles for perioperative care.

## Figures and Tables

**Table 1:** Comparison of the 213 laparoscopic-treated patients with 327 patients treated with conventional open procedure in the same period (November 2004–December 2008) in our department.

Parameter	Laparoscopic procedure	Conventional open procedure	*P* value
	*n* = 213	*n* = 327	
Sex (male/female)	114/99	158/169	ns

Age (years, median (range))	70 (36–94)	72 (36–92)	0.002

BMI (kg/m^2^, median (range))	23.9 (16.4–42.3)	23.4 (13.9–36.0)	0.023

ASA class (%)			
I	32 (16)	34 (11)	
II	145 (70)	218 (69)	ns
III	30 (15)	62 (20)	
IV	0 (0)	3 (1)	

Blood loss (mL, median (range))	50 (0–1600)	200 (0–2700)	<0.001

Number of patients with a primary anastomosis (%)	184 (86)	237 (72)	ns

Number of lymph nodes in resection (median (range))	15 (2–50)	15 (1–75)	ns

Hospital stay (days, median (range))			
With stoma	10 (5–24)	10 (5–14)	ns
With primary anastomosis	4 (1–55)	8 (2–109)	<0.001
Total	5 (1–55)	8 (2–109)	<0.001

Clavien-Dindo Classification of Surgical complications (%)			
No complications	168 (79)	223 (68)	0.006
Grade I	7 (3)	14 (4)	
Grade II	9 (4)	32 (10)	
Grade III-a	2 (1)	3 (1)	
Grade III-b	24 (11)	35 (11)	
Grade IV-a	2 (1)	3 (1)	
Grade IV-b	0 (0)	0 (0)	
Grade V	1 (0)	17 (5)	

Dukes classification (%)			
Dukes A	14 (7)	24 (7)	
Dukes B	106 (50)	148 (45)	
Dukes C	59 (28)	93 (28)	
Dukes D	28 (13)	47 (14)	
Not classified	6 (3)	15 (5)	

BMI: body mass index, ASA: American Society of Anaesthesiology, ns: nonsignificant (*P* > 0.05).

**Table 2:** Operative procedures.

Operative procedure	*n* (%)
Laparoscopic (*n* = 213)	
Sigmoid resection	72 (34)
Sigmoid resection with stoma	2 (1)
Left hemicolectomy	17 (8)
Right hemicolectomy	40 (19)
Transversum resection	2 (1)
Low anterior rectal resection	47 (22)
Low anterior resection with stoma	9 (4)
Hartmanns procedure	9 (4)
Abdominoperineal resection	6 (3)
Colectomy	3 (1)
Other	6 (3)

Open (*n* = 327)	
Sigmoid resection	27 (8)
Sigmoid resection with stoma	2 (1)
Left hemicolectomy	30 (9)
Left hemicolectomy with stoma	1 (0)
Right hemicolectomy	143 (44)
Transversum resection	4 (1)
Colectomy	7 (2)
Low anterior rectal resection	25 (8)
Low anterior resection with stoma	13 (4)
Hartmanns procedure	53 (16)
Abdominoperineal resection	14 (4)
Other	8 (2)
